# Organizational Conditions That Impact the Implementation of Effective Team-Based Models for the Treatment of Diabetes for Low Income Patients—A Scoping Review

**DOI:** 10.3389/fendo.2020.00352

**Published:** 2020-07-15

**Authors:** Maria Levis-Peralta, Maria del Rosario González, Renée Stalmeijer, Diana Dolmans, Jascha de Nooijer

**Affiliations:** ^1^Impactivo, LLC, San Juan, Puerto Rico; ^2^Department of Educational Development and Research, School of Health Professions Education, Maastricht University, Maastricht, Netherlands; ^3^Department of Health Promotion, School of Health Professions Education, Maastricht University, Maastricht, Netherlands

**Keywords:** team-based care, organizational conditions, interprofessional collaborative practice, diabetes, low-income

## Abstract

**Background:** Team-based care models (TBC) have demonstrated effectiveness to improve health outcomes for vulnerable diabetes patients but have proven difficult to implement in low income settings. Organizational conditions have been identified as influential on the implementation of TBC. This scoping review aims to answer the question: What is known from the scientific literature about how organizational conditions enable or inhibit TBC for diabetic patients in primary care settings, particularly settings that serve low-income patients?

**Methods:** A scoping review study design was selected to identify key concepts and research gaps in the literature related to the impact of organizational conditions on TBC. Twenty-six articles were finally selected and included in this review. This scoping review was carried out following a directed content analysis approach.

**Results:** While it is assumed that trained health professionals from diverse disciplines working in a common setting will sort it out and work as a team, co-location, and health professions education alone do not improve patient outcomes for diabetic patients. Health system, organization, and/or team level factors affect the way in which members of a care team, including patients and caregivers, collaborate to improve health outcomes. Organizational factors span across seven categories: governance and policies, structure and process, workplace culture, resources, team skills and knowledge, financial implications, and technology. These organizational factors are cited throughout the literature as important to TBC, however, research on the organizational conditions that enable and inhibit TBC for diabetic patients is extremely limited. Dispersed organizational factors are cited throughout the literature, but only one study specifically assesses the effect of organizational factors on TBC. Thematic analysis was used to categorize organizational factors in the literature about TBC and diabetes and a framework for analysis and definitions for key terms is presented.

**Conclusions:** The review identified significant gaps in the literature relating to the study of organizational conditions that enable or inhibit TBC for low-income patients with diabetes. Efforts need to be carried out to establish unifying terminology and frameworks across the field to help explain the relationship between organizational conditions and TBC for diabetes. Gaps in the literature include research be based on organizational theories, research carried out in low-income settings and low and middle income countries, research explaining the difference between the organizational conditions that impact the implementation of TBC vs. maintaining or sustaining TBC and the interaction between organizational factors at the micro, meso and macro level and their impact on TBC. Few studies include information on patient outcomes, and fewer include information on low income settings. Further research is necessary on the impact of organizational conditions on TBC and diabetic patient outcomes.

## Introduction

The prevalence of type 2 diabetes has increased worldwide from 4.7% in 1990 to 8.5% in 2014 becoming a major global public health issue that affects 422 million people (1). In the United States alone, 30 million patients have diabetes (9.4% of the population). According to the American Diabetes Association (2), the estimated total economic cost of diagnosed diabetes in 2017 was $327 billion. Statistics in Canada and the UK reflect 2.3 million (6% of the population) and 4.7 million (7% of the population) people affected, respectively (1). These countries have directed significant resources toward addressing type 2 diabetes, which is largely preventable. However, prevalence continues and is on the rise (3–5).

Diabetes disproportionately affects middle-and low-income countries with 77% of the world's diabetes patients living in these Nations (4). In China, diabetes has been considered a burden to the health system with a prevalence of 11.6% in 2010 (6). The International Diabetes Federation has estimated that there are about 32.8 million people with diabetes in the Middle East and North Africa. For example, in Oman it is estimated there is going to be a three-fold increase of diabetes patients in the next years (7).

Additionally, people living in low income and disadvantaged communities have a higher prevalence of type 2 diabetes than the general population (8). This correlation has been found in studies where, after controlling for age and education, regression models have confirmed an association of low income with an increased prevalence of the condition and its complications (9). Low socioeconomic status has also been “associated with almost a two-fold risk” of diabetes-related mortality and disparities remain even after controlling for risk factors (10).

Patients with type 2 diabetes and other chronic complex conditions require a coordinated, comprehensive and collaborative care approach (11). This becomes even more necessary when patients have limited resources and require additional support accessing food, transportation, and other non-medical factors. Additionally, primary care practices in low resourced settings face constant pressures to address more—*complex population, treatment complexity, regulations, health expenditures*—with less—*resources, payments, health professionals*. Furthermore, it is at the primary care level that health promotion, prevention interventions, health education and anticipatory guidance related to diabetes are addressed in an environment where attention must also be given to the social determinants of health. Practices and healthcare systems are aware that no matter the challenges, primary care needs to transform if they aspire to provide quality care (12).

Team-based care (TBC) has demonstrated effectiveness to improve health outcomes for diabetes patients and provide higher quality care (13). TBC is defined by the National Academy of Medicine as “the provision of health services to individuals, families, and/or their communities by at least two health providers who work collaboratively with patients and their caregivers—to the extent preferred by each patient—to accomplish shared goals within and across settings to achieve coordinated, high-quality care” (14). In this study, we focus specifically on TBC that happens at a primary care setting where health professionals from various disciplines are engaged in care to improve diabetes outcomes. TBC has also been found to increase the satisfaction and productivity of primary care practices (15). TBC is particularly important for under-resourced settings because it results in expanded access to care, more efficiency in the use of limited resources, reductions in care fragmentation, and comprehensive patient-centered care where each member of the team renders services interdependently avoiding duplication (16–19). TBC has been considered a way to increase quality and strengthen the healthcare systems (20). Interprofessional Collaboration (IPC) is a key element of TBC where “multiple health workers from different professional backgrounds provide comprehensive services by working with patients, their families, careers, and communities to deliver the highest quality of care across settings” (21). Therefore, for the purpose of this study, the terms are used interchangeably as an equivalent concept (22).

Specifically related to diabetes, TBC is associated with statistically significant reductions in HbA1c values which are larger than those of other quality improvement strategies (23). A systematic review and meta-analysis of 48 cluster RTC and 94 patients RCT on the effectiveness of interventions in the management of diabetes found that a significant mean decrease in HbA1c over a median follow-up of 12 months had the strongest positive association with TBC (13). Another meta-analysis of cluster and randomized controlled trials revealed that TBC achieved the best patient outcomes with respect to control of diabetes, particularly those with the poorest outcomes at baseline (24). Other studies have also demonstrated that people with diabetes can experience improved clinical outcomes when their care is provided locally through TBC (11), including decreases in patient's use of medications, morbidity mortality, utilization and cost, while increasing self-management and empowerment, healthy behaviors, satisfaction, and quality of life as well as increased job satisfaction (25). A recent study also found that socioeconomically diverse adults with type 2 diabetes believe that a coordinated team based care was a “good approach” suggesting its capacity to address socioeconomic challenges faced by low income patients (26). Additionally, a study of factors influencing type 2 diabetes self-management found that medically underserved patients who are able to manage their condition described a TBC approach of care and support (27).

Although there is significant evidence that TBC in primary care settings is beneficial for patients with diabetes, TBC interventions are not always able to demonstrate improvements in quality of care (28). In general terms, it is assumed that by placing health professionals from diverse disciplines together, somehow, they will sort it out and start working as a team. Nothing is farthest from reality. Even when health professionals are co-located in the same facility and express willingness and confidence to engage in TBC, studies have found that they express that they do not have the resources to fully implement TBC for diabetic patients (7). However, few studies have identified which factors are required to organize teams in primary care settings to effectively prevent and manage diabetes.

Various studies have identified organizational conditions as influential on TBC, however, the organizational factors that enable and inhibit TBC have not been well-defined and studied. A recent research review synthesizing the “existing evidence and theory on the science of healthcare teams and healthcare team training” identified the understanding of organizational conditions as an area where future research needs to be developed to improve our understanding of health teams (29).

Therefore, it is important to synthesize the state of the literature on the organizational conditions that impact TBC in primary care settings to obtain better insights into what aspects of the organizational conditions influence TBC and improved diabetes outcomes and what deserves future attention. While the concept of organizational conditions is discussed in various articles, it has yet to be defined. We therefore proposed the following definition:

“*Organizational conditions are the health system, organization and/or team level factors that affect the way in which members of a care team, including patients and caregivers, collaborate to improve health*.”

As specified in the definition, these factors can be found at different levels within an organization, the macro-level (i.e., the health system), the meso-level (i.e., the organization), and the micro-level (i.e., the team). For this reason, we used the ecologic model as an umbrella for the review, in which macro, meso, and micro environments are the starting point for what the literature says about how team-based diabetes care is organized. It is also important to consider the implications of alignment or lack of alignment between these factors across levels and its implications.

Governments worldwide are carrying out efforts to prevent and improve diabetes outcomes, particularly in low income communities where the condition is most prevalent. Although TBC shows significant potential for improving diabetes outcomes, researchers have identified organizational factors as potentially mediating TBC and its effectiveness. The purpose for conducting this scoping review is to identify themes in the literature and analyze knowledge gaps with respect to the topic of organizational conditions that enable or inhibit TBC for diabetic patients in primary care settings. This scoping review aims to answer the question: What is known from the scientific literature about how organizational conditions enable or inhibit team-based care for diabetic patients in primary care settings, particularly settings that serve low-income patients? In answering this research question our aims are to:

Aim 1: Identify what organizational factors have been cited in the existing literature as enabling and/or inhibiting TBC for diabetes care in primary care settings and categorize these factors.
Aim 2: Identify how the literature describes the relationships between organizational factors and TBC for low income patients.

## Methods

### Study Design

To address the research question and aims a scoping review study design was selected because it allows for a rapid gathering of the literature in a particular area to identify key concepts and research gaps (30). This scoping review was carried out following the approach of directed content analysis of studies retrieved from diverse sources (31). This research method is useful for “classifying large amounts of text into an efficient number of categories that represent similar meaning” (32). The scoping review protocol used for this study followed the five stages of the Asksey and O'Malley framework (33):

1. Identifying the research question.
2. Identifying relevant studies (search strategy).
3. Study selection.
4. Charting the data.
5. Collating, summarizing, and reporting results.

### Identifying Relevant Studies (Search Strategy)

Our team performed a search using NCBI PubMed and MEDLINE A Boolean keyword search was performed to include four different concepts: “Team-Based Care” AND “Diabetes” AND “Organizational Conditions” AND “Low-Income.” The search was conducted using the four mentioned concepts and combinations of associated relevant keywords, either synonyms or alternative terms for each of these concepts: “Team-based Care” (OR “Team-based” OR “Interprofessional Collaborative Practice” OR “Interprofessional”) AND “Diabetes” AND “Organizational Conditions” (OR “Organizational Context” OR “Organizational” OR “Organizational Factors”). The study selection was performed systematically to provide a broad scope of the literature available with the aim of being inclusive.

The search was performed for articles published before June 20, 2019 written in the English language using all dates available in the database. In addition, we scanned the reference lists of articles for relevant published studies using what is called snowballing and reverse snowballing (34). This search produced 132 articles.

### Studies Section

The bibliographic information of the studies resulting from the database searches were downloaded and organized. Two researchers screened the article abstracts to determine initial eligibility against inclusion/exclusion criteria described below.

1. Published in English.
2. Focused on care teams that include a primary care physician and at least two health professionals from different disciplines.
3. Focused on teams working in primary care settings.
4. Discussion of the relationship between organizational conditions and TBC for diabetes patients.
5. Discussion of the organizational factors and/or conditions that improve outcomes for diabetes patients.
6. Qualitative, quantitative studies, and reviews.

Articles were excluded for the following reason.

1. Not substantiated by theory and/or research findings; Articles considered not to be substantiated by theory and/or research findings include: papers that were not peer reviewed, opinion pieces, and articles that did not use a research methodology to arrive at findings.
2. Did not include information about organizational factors, conditions, context, and/or supports.
3. Care teams do NOT include a primary care physician.
4. Care teams are NOT multidisciplinary.
5. Full article was not available.
6. Not pertinent to diabetes.

**Figure 1:**
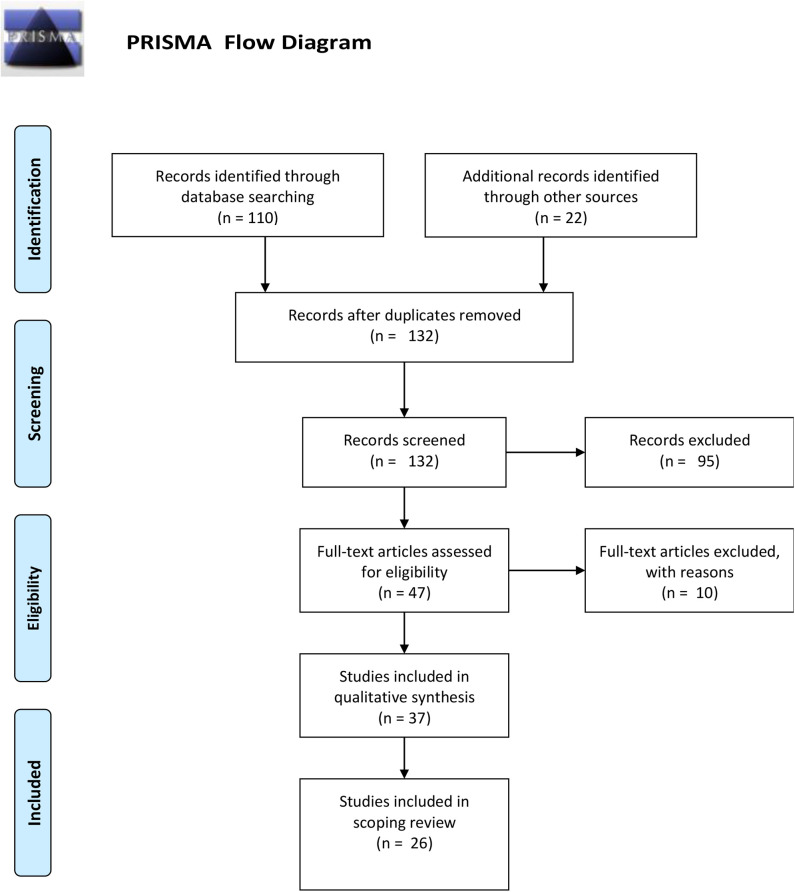
PRISMA flow diagram.

Any discrepancies between the determinations of the two researchers were discussed for consensus. Full texts of these articles were then read by two researchers (ML and MG) and charted for further confirmation of inclusion. Figure 1 shows the PRISMA Flow Diagram detailing the process. Twenty-six articles were finally selected and included in this review.

### Charting the Table (Analysis)

Selected articles were reviewed and full articles read in detail by two researchers. Data collected from the studies was charted in a data-charting form using a directed content analysis method. Content categories include:

- a full reference citation (author, title, journal, and date published).
- the level of the organization/community impacted (micro-level/the team, meso-level/the organization, or macro-level/the system).
- the methodology used by researchers.
- a summary of key characteristics or organizational factors and findings.
- if the study includes data specific to low income populations.
- the impact on patient outcomes studied (if available).

One researcher proceeded to extract the data from the articles. Once an initial draft was available, another researcher reviewed the data extractions against the full-text articles for alignment, and discrepancies were discussed. This process of extraction was carried out iteratively and refined based on discussions by the entire research team. The coding structure was applied to all the articles to identify all instances of these themes. All researchers had access to and reviewed the data charting form. A summary is presented as Table 1.

**Table 1:** Summary of literature reviewed.

**Author name**	**Methodology used**	**Cited impact on patient outcomes**	**Data specific to low income setting**	**Organization/community level**	**Categories mentioned**
Ackroyd and Wexler (24)	Review	Positive impact	N/A	Micro/Meso	Technology, workplace culture, structure and processes, team skills, and knowledge, resources
Al-Alawi et al. (7)	Qualitative research	Not reported	Yes	Micro/Meso	Team skills and knowledge, technology, workplace culture, financial implications, structure and processes, and resources
Black et al. (28)	Randomized control trial	Reported, but not significant	Yes	Micro/Meso	Financial implications, structure and processes, technology, team skills, and knowledge
Foster et al. (35)	Qualitative research	Not reported	No	Micro/Meso	Workplace culture, policies, structure and processes, resources
Gucciardi et al. (36)	Qualitative research	Not reported	No	Micro	Team skills and knowledge, workplace culture, governance, structure and processes, and resources
Hepworth et al. (37)	Qualitative research	Not reported	No	Micro	Resources
Kaufman et al. (38)	Qualitative research	Positive impact	Yes	Meso/Macro	Financial implications, structure and processes, technology, and governance
Li et al. (6)	Mixed methods	Positive impact	Yes	Macro	Financial implications, structure and processes, technology, policies governance, structure and processes, and resources
Lublóy et al. (39)	Mixed methods	Positive impact	No	Micro	Workplace culture and resources
MacLeod et al. (40)	Qualitative research	Not reported	No	Meso	Structure and processes, team skills and knowledge, workplace culture, and resources
Manns et al. (41)	Qualitative research	Not reported	No	Micro/Meso/Macro	Structure and processes, workplace culture, financial implications, and resources
McDonald et al. (42)	Mixed methods	Not reported	No	Micro/Meso	Financial implications, structure and processes, team skills and knowledge, workplace culture, financial implications, governance, and resources
McDonald et al. (43)	Mixed methods	Not reported	No	Micro, Meso Macro	Structure and processes, workplace culture, governance, and resources
Mur-Veeman et al. (44)	Qualitative research	Not reported	No	Meso	Structure and processes, workplace culture
Nagelkerk et al. (45)	Mixed methods	Positive impact	Yes	Micro	Structure and processes, team skills and knowledge, and resources
Noël et al. (46)	Randomized control trial	Positive impact	Yes	Micro	Team skills and knowledge, technology, and workplace culture
Price-Haywood et al. (47)	Qualitative research	Reported, but not significant	Yes	Meso	Structure and processes, technology, workplace culture, and resources
Quinn et al. (48)	Mixed methods	Positive impact	No	Micro	Structure and processes, workplace culture, structure and processes, and resources
Raaijmakers et al. (49)	Qualitative research	Not reported	No	Meso	Financial implications, structure and processes, technology, workplace culture, team skills and knowledge, and resources
Russell et al. (50)	Mixed methods	Positive impact	Yes	Macro	Financial implications and resources
Siriwardena et al. (51)	Qualitative research	Not reported	No	Meso	Financial implications, structure and processes, team skills and knowledge, governance, and resources
Tomoaia-Cotisel et al. (52)	Review	Not reported	No	Meso/Macro	Financial implications, structure and processes, technology, governance, team skills and knowledge, and resources
Van der Wees et al. (53)	Mixed methods	Not reported	Yes	Micro/Meso	Structure and processes, governance, workplace culture, and resources
Van Eeghen et al. (54)	Qualitative research	Reported, but not significant	No	Meso	Structure and processes, and resources
Watts et al. (55)	Qualitative research	Not reported	No	Meso	Technology and resources
Yu et al. (56)	Qualitative research	Not reported	No	Meso/Macro	Structure and processes, team skills and knowledge, technology, structure and processes, workplace culture, and resources

### Collating, Summarizing, and Reporting Results

The first author developed a thematic map with this data to organize the information that answers the research question in a way that it provides an overview of what is available in the literature but not a synthesis. Data related to the extent and nature of the studies is summarized using tables and charts and a thematic analysis is presented. This process entailed developing thematic coding which emerged from the literature. This data was synthesized and presented to the research team for discussion through an iterative process. The team consists of clinicians, medical educators, and health system's researchers. The results presented represent the analysis ensuing from this process.

## Results

### Descriptive Summary

The studies included in this review spanned the USA, Canada, Europe (UK and Netherlands), Asia (China), and the Middle East (Oman). Methodologically, these studies were principally qualitative (7, 35–38, 40, 41, 44, 47, 49, 51, 54–56), with a few using mixed methods (6, 39, 42, 45, 48, 50, 53) and two randomized control trials (28, 46). Furthermore, two reviews (24, 52) were included about diabetes that provided some insights into the organizational context necessary for team-based care, although they were not specifically about organizational conditions.

The included studies did not use unified terminology when addressing organizational conditions. Instead, these studies focused on TBC for diabetes and discussed several aspects of the implementation which included organizational factors. Only one study looked specifically at the impact of organizational factors on TBC for diabetes (50). In the rest of the studies, organizational factors are identified tangentially and were not the focus of the research. Most of the research focused on identifying the factors that were present in practices which resulted in TBC for diabetes (24). Therefore, there is practically no empirical data on the effect of the organizational factors cited on TBC for diabetes (6, 7, 26, 35–37, 39, 43–46, 48, 51, 53, 57, 58). It is important to note that the three studies that did not see improved outcomes had a focus on Structure and Processes (the only unifying factor) and none focused on Governance (28, 47, 54).

Additionally, while studies discuss enabling factors to TBC that are specific to the organization, the term “organizational conditions,” has yet to be defined. For the purpose of this study, we have defined organizational conditions as the health system, organization and/or team level factors that affect the way in which members of a care team, including patients and caregivers, collaborate to improve health. Organizational conditions include factors that affect governance and policies, structure and process, workplace culture, resources, team skills and knowledge, financial implications, and technology of the setting in which TBC is implemented.

While the analysis identified that the literature on this topic is extremely dispersed with no unifying terminology, studies do analyze enablers to TBC according to different levels of the organization which are impacted. Additionally, key concepts emerged and have been categorized through a thematic analysis.

### Organizational Factors Related to TBC for Diabetes Care

The first aim of this study was to identify what organizational factor have been cited in the existing literature as related to TBC for diabetes care in primary care settings and categorize these factors according to the level of the organization impacted and key concepts.

#### Level of Analysis

An important finding is that organizational factors identified in the literature spanned three levels of analysis.

- Macro-level: Focusing on health systems and factors that impact multiple organizations.
- Meso-level: Focusing on the primary care organization from a management perspective and factors that impact multiple care teams within the organization.
- Micro-level: Focusing on factors that impact the patient's immediate care team and its members, including the patient.

Further understanding organizational factors at each level of analysis can help delineate the focus area of the study, how decisions are made within organizations, and the subjects impacted by the factors as detailed in Table 2.

**Table 2:** Levels of analysis.

	**Macro-level**	**Meso-level**	**Micro-level**
Focus	Health system	Health organization	Patient care team
Impact	Multiple health organizations	Multiple patient care teams	Care team members, including the patient
Decision makers	Government authorities, payers, technology providers	Organization's management team, board of directors	Care team formal and informal leaders, including the patient

While all the levels of analysis impact the care team members, depending on the health system, organizational factors could be assigned to a specific level based on who in the organization is responsible for making the decisions related to each factor. At the macro-level government authorities, payers, or technology providers make decisions related to the factors. At the meso-level, the organization's management team or board of directors make the decisions and at the micro-level, the team care team, including the patient, has the authority to make the decisions regarding the factors. The levels of analysis are an important distinction in that it provides a framework for understanding who is responsible for enabling factors and therefore addresses concerns by health professionals and the deployment of resources to engage in TBC (7). Although most studies focused their analysis on one of these levels, various studies blurred the boundaries between the levels. It is noteworthy that these studies were mostly qualitative in which the focus of study was the team (micro-level) and the team members identified the other levels (41, 53), even though they do not exercise direct control over these factors. Further research also needs to go into understanding how decision-making plays a role in the organizational conditions and which factors in each level have different effects on TBC for diabetic patients.

#### Key Concepts

This study aims to identify what organizational factor have been cited in the existing literature as enabling and/or inhibiting TBC for diabetes care in primary care settings and categorize these factors. The studies included in this review present an extremely dispersed understanding of which organizational factors enable TBC with no unifying terminology or agreement on which are required. Additionally, no information was provided in the studies about the organizational factors that inhibit TBC. However, the direct content analysis revealed that the organizational factors which impact TBC could be organized into seven principal categories (see Table 3):

**Table 3:** Organizational factors categories according to level of analysis.

**Categories**	**Macro-level**	**Meso-level**	**Micro-level**
Governance and policies	✓	✓	✓
Structure and processes	✓	✓	✓
Workplace culture		✓	✓
Resources		✓	✓
Team skills and knowledge		✓	✓
Financial implications	✓	✓	✓
Technology	✓	✓	✓

Governance and policies, financial implications, and technology cut across all organizational levels of analysis. It is important to note that these categories that cut across all levels of analysis all include factors which are outside the control of teams and their organizations. This is important because organizational conditions at the Macro-level may be able thwart TBC or conversely Meso and Micro-level organizational factors may be able to mediate for macro-level factors. For example, different meso and micro-level organizational factors may impact TBC under different Macro-level financial implications under a fee for service, capitation, or staff model payment policy. All other categories focused on the meso and micro-level of analysis.

We do not posit that synthesized categories are exhaustive or exclusive, but these provide further insights into how organizational factors enable and inhibit TBC. Further information to define the key concepts is provided below:

#### Governance and Policies (GP)

Governing and governance refers to the way that health systems are governed, and decisions are made at all levels of analysis. Specifically, this GP category includes Macro-level factors that impact TBC, including government policies that impact social services, nutrition and physical activity, the organization's participation in cross-cutting organization arrangements (including integrated service delivery models that coordinate care across various clinical settings), membership in coalitions that span multiple organizations (including patient networks which provide expanded supports to patients and multi-sector coalitions/coordination that transcends health services and address social determinants of health) (6, 38, 50). At a meso and micro level, the establishment of mechanisms for accountability and compliance with the course of action adopted at a policy level are cited (42). However, the studies did not address the process of decision-making or levels of authority within the organizations. We were also unable to ascertain if there were any commonalities between the governance of different systems across countries.

#### Financial Implications

This category was one of the most cited and cut across all three levels of analysis. It focuses on the monetary resources necessary to run the organization (dictionary.com). At a macro-level factors cited include the availability of financial incentives for provider and patients (including pharmacy benefits) and the total cost/budget as a binding constraint. At a meso-level, the literature focused primarily on the organization's capacity to maximize billing so as to offset costs related to TBC and the models that exist for staff compensation (for example per patient per month, salaried, and hourly). On a micro-level, the principal focus was on team member's understanding and access to information on the financial implications of action on the practice (6, 7, 28, 38, 41, 42, 49–52). There was very limited data on how financial implications play out in low-income settings or social medicine.

#### Technology

Factors for this category permeated all three levels of analysis and the focus was primarily on health information technology. At a macro-level studies identified organizational factors focused on the capacity to exchange clinical and billing data across providers to improve coordination. Significant attention is also provided to the role of technology at the meso level including access to an electronic medical record (EMR), a data manager, technical resources, data analytics capacity, patient registries, hospital admissions data, compliance, and quality metrics (6, 7, 24, 28, 38, 46, 47, 49, 52, 55, 56). At the micro-level a U.S. Agency for Healthcare Research and Quality review (59) on the implementation of its diabetes care models provides significant guidance related to technology supports for the team citing EMRs and IT that enable and support “communication among care team members (e.g., via electronic patient care plans); managing patient registries; coordinating, and documenting services to be delivered in a structured manner; reminding patients about appointments or contacting patients who were lost to follow-up; creating and tracking patients' plans of care and progress toward their goals; identifying additional “triggers”.” Further research needs to be carried out in low income settings countries and communities with low income patients.

#### Structure and Processes (SP)

Organizational factors cited which were grouped in this category refer to formal organizational rules, documentation, and processes which define the relationships between different actors within organizations and the action steps taken by team members to achieve a particular end. These factors tend to guide the operations of organization and teams and are focused at the meso and micro level.

SP factors identified at a meso-level in the literature include:

- Definition of roles, responsibilities, and job descriptions (40, 51).
- Establishments of workflows (54).
- Care coordination across providers, continuity of care, and synchronizing of services (44).
- Practice based linkages to community services to address social determinants of health (38).
- Communication mechanisms and meetings (49).
- Time for organization, collaboration, and learning (51).
- Patient appointment systems (28, 52).
- Quality improvement infrastructure (13, 52, 55, 60).
- Issues related to the location of resources and the physical space where services are provided (6, 27, 42).

SP factors identified at the micro-level focused specifically on the establishment of workflows, systems for improving communication (including meetings and reminders), carrying out quality improvement activities and using benchmarking data (36, 45, 48).

#### Workplace Culture (WC)

This category includes the customs and behaviors carried out at the meso and micro-level that impact TBC (dictionary.com). At a meso level WC factors include leadership, power dynamics, and morale (40, 44, 47, 49).

Workplace culture is represented at the micro level through a broad spectrum of factors in addition to those cited above, including trust, team climate, strength of relationships, motivation/will, role perception, perceived benefit, flexibility for implementation, and shared between team members (36, 39, 46, 48).

#### Team Skills and Knowledge

Organizational factors related to team member's competence to carry out TBC and their access to information necessary to carry out their role in the team. At a meso-level, the literature focused on the capacity of management to structure opportunities for formal training, informal learning, and access to information (40, 49, 51).

On the micro-level, factors focused on team member's capacity for reciprocal learning, demonstrated competence and knowledge acquisition. Specific emphasis was given in the literature to the role of the patient education, as a member of the care team, and their skills with relation to self-management (36, 45, 46).

#### Resources

Resources including money, material and staff that team members and patients can draw down to carry out their functions and purpose were cited as organizational conditions that needed to be present for TBC. Most of the factors in this category were identified at the meso-level since this is where they tend to be deployed and/or distributed, however, further research needs to identify who makes decisions about these in different settings and if it has an impact on TBC. The factors cited at this level include the team's composition, the human resource recruitment process, caseload optimization, practice sizes, and patient to physician rations. It is important to note that with respect to team composition the literature on TBC for diabetes spanned a broad network of multi-disciplinary team members which could collaborate with the primary care physician. Potential team members cited in the literature include Specialists, Registered Nurses, Nurse Practitioners, Nutritionist, Physical Therapist, Mental Health Providers, Diabetologists, Pharmacists, Community Health Workers, Care Managers, Health Coaches, Health Managers, Diabetes Nurses, and Medical Assistants (40, 47, 49, 51, 54, 55). At the micro-level, the human resource availability and stability were cited as important factors (36, 37, 39, 45, 48).

### Relationships Between Organizational Factors and TBC for Low Income Patients

The second aim of this study is to identify how the literature describes the relationships between organizational factors and TBC for low income patients. Close to a third (6, 7, 28, 38, 45–47, 50, 53) of the studies included in the review provide information that includes low income/low resource settings. Studies come from China, Oman, Australia, Canada, and the United States. These include randomized control trials (28, 46), four mixed methods (6, 45, 50, 53), and three qualitative studies (7, 38, 47). Most of these studies cited the impact of workplace culture, resources, structure, and processes as factors impacting TBC for low income/low resource settings.

Five of these studies discussed diabetes patient outcomes for low income/resource settings. Out of these studies, most saw significant increases in the quality of care for diabetic patients including reductions in emergency room visits, decreased hospital admissions, increased clinic efficiency, improved clinical laboratory results (including Hg A1C Control) and improved general health, among others (6, 38, 45, 50). These studies were mainly focused on a macro-level analysis, financial implications, and policy recommendations. One study which was evaluating the implementation of TBC for the purpose of improving diabetes outcomes in a low resourced setting saw no impact relating to TBC (28). The intervention implemented addressed organizational factors focused on financial implications, technology, structure and process, and team skills and knowledge, but did not address governance, workplace culture, or resources.

While a few studies address patients in low-income settings, more research needs to be carried out. It is important to note that there was scant literature in low-income countries, and no research in any of the countries in Africa and Latin America. Additionally, only one study took into account the patient's social determinants of health (poverty, literacy, housing, citizenship, and others) even though the capacity to address these can be enabled through TBC.

## Discussion

### Findings

This article set out to review what is known from the scientific literature about how organizational conditions enable or inhibit TBC for diabetic patients in primary care settings, particularly settings that serve low-income patients, and identify directions for further research. Our first finding is that even though TBC has significant evidence supporting its effectiveness on diabetes outcomes, co-location of healthcare professionals, and providing clinician education alone do not improve patient outcomes for diabetic patients. Organizational conditions are a pre-requirement to effective TBC (28, 47, 54). While significant evidence points to the potential of TBC to impact diabetic patient outcomes (13, 24, 60), there is still a very limited understanding of the organizational factors that enable and inhibit it. Although various articles have cited the need to understand the organizational conditions that enable TBC (29), the literature does not define the term organizational conditions, nor does it provide frameworks for analyzing these conditions. This paper has defined the term organizational conditions as the health system, organization, and/or team level factors that affect the way in which members of a care team, including patients and caregivers, collaborate to improve health. Consensus needs to be reached with respect to a working definition for the term “organizational conditions” so as to better define an area of study.

Second, our research found that factors that impact TBC span various organizational levels of analysis and future studies need to address the topic of who and how decisions are made related to the organizational factors that impact TBC. This is an important distinction in the study of organizational conditions that can help explain why similar interventions may not have the same results (28). For example, the financial implications of TBC are an often-cited in the literature as a barrier to TBC (7, 41, 42), but organization using different payment models have been able to achieve TBC. An analysis by organizational level can help explain if changes in financial implications at the Macro, Meso, or Micro-level are required or if there is a necessary alignment between them which impacts TBC. Additionally, in different countries and health systems the organizational factors are enabled or disabled at a different label leading to potentially different results. For example, in some organizations teams at the micro-level are responsible for scheduling meetings and in others these are scheduled at the meso-level with potentially different results.

**Figure 2:**
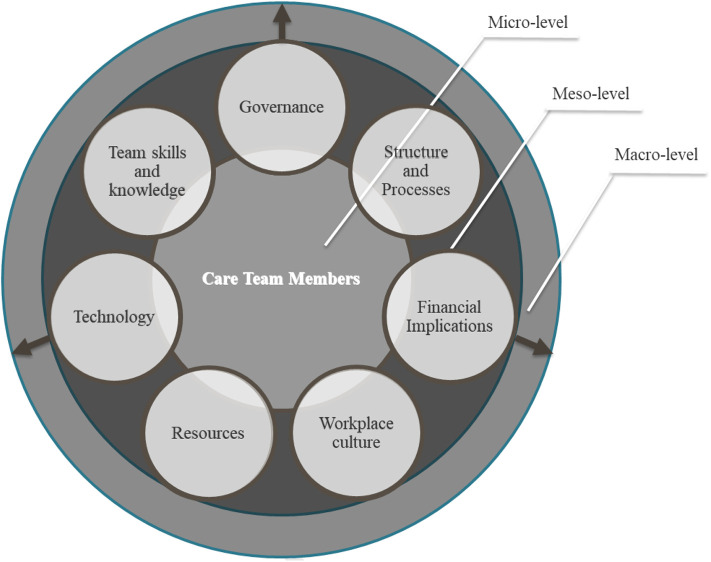
Framework for analysis of organizational factors that impact team-based care.

Third, the dedicated content analysis demonstrated how the included studies cite numerous factors external to the care team as influencing TBC to improve diabetes outcomes, however, the literature on this topic is extremely disperse with no theoretical frameworks used to understand the phenomenon across studies. This is the first paper that seeks to categorizes the organizational factors related to TBC for each level of analysis (see Figure 2). Governance, financial implications, and technology included factors that spanned all three levels of analysis, while the rest of the factors focused exclusively on the micro and meso levels. Further research needs to look into which of the factors within these categories are pre-requisites, enablers, and/or inhibiters of TBC and at what level of the organization.

Finally, the review identified significant gaps in the literature relating to the study of organizational conditions that enable TBC for diabetes prevention and management to address the needs of low-income patients. Gaps in the literature include research in low-income countries (particularly countries in Africa and Latin America), research on governance, workplace culture and/or resources, and models of TBC that address patient's social determinants of health.

TBC implies a shift from the traditional healthcare model based on the physician assuming all responsibility for patient care to a model of care where multiple health professionals work with patients and families share responsibility over patient outcomes. While, several publications have addressed the challenges faced and processes of transitioning from traditional care to TBC (59), few have focused specifically on TBC for diabetes management and even less have focused on low resource settings creating a significant gap.

There is a strong need for studies that explore which categories or factors are more highly associated with TBC that is able to achieve improved diabetes patient outcomes for low income patients.

### A New Research Agenda

We suggest future research on how organizational factors present at each level of analysis in primary practices that demonstrate effectiveness in patient diabetes control vs. those who show poor outcomes. Additionally, more design-based research (reference) is necessary to demonstrate the replicability of improved patient outcomes by implementing organizational conditions for TBC. Questions that still need to be answered include:

- How do primary care team members (micro) that have been able to demonstrate improved diabetes patient outcomes in primary care for low income patients perceive and define their organizational conditions for TBC? How do these differ from organizations that co-locate health professionals and demonstrate poor diabetes outcomes?
- Which organizational conditions can be found at primary care practices that have been able to demonstrate improved patient outcomes in primary care for low income patients from the perspective of the organization's management (Meso) vs. care teams (Micro)? How do these differ from organizations that co-locate health professionals and demonstrate poor diabetes outcomes?

These and others are questions for future efforts in the implementation of a transformational process that will continue for years to come.

### Limitations

This study has various limitations due to the lack of existing research, unifying terminology, and frameworks on organizational conditions that enable TBC for diabetes prevention and management. Most studies included in this review discussed organizational factors which were present when TBC was successful, but none of the studies included any information on which organizational factors inhibit TBC. Additionally, none of the studies provided any effect sizes relating to which of the organizational factors were a requirement for effective TBC. The studies also tended to blend the different organizational levels of analysis and none of the studies specifically discussed the process of making decisions regarding the organizational factors. Information was even more limited for primary care practices serving low income patients. It is also important to note that few studies were published in other languages or in low-income countries which limits the analysis to health systems that have infrastructure similar to those in the United States and Europe. Therefore further research needs to be carried out in order to assess is these organizational factors translate to other regions or cultures.

## Conclusions

The review identified significant gaps in the literature relating to the study of organizational conditions that enable or inhibit TBC for low-income patients with diabetes. Efforts need to be carried out to establish unifying terminology and frameworks across the field to help explain the relationship between organizational conditions and TBC for diabetes. Less than a third of studies included in the review (11 studies) include some measure of the impact on patient outcomes. Most of the studies provided observations from practices that had demonstrated an improvement on patient outcomes after implementing TBC. However, no trends could be identified relating to which organizational factors were more prevalent in these studies. Fewer studies include information on low income settings. The researchers found that few publications specifically addressed the organizational factors identified in the study which presents an opportunity to promote additional research. The researchers also recommend that future research be based on organizational theories, such as Engstrom's Activity Theory (1987), Pettigrew and Whipp's Dimensions of Change (1992), or Ferlie and Shortell's Framework for Change (2001) to delve deeper into the inter-relation of the organizational factors. It would also be useful to understand if there is a difference between the organizational conditions that impact the implementation of TBC vs. maintaining or sustaining TBC. It would also be important for future studies to include the interaction between organizational factors at the micro, meso, and macro level and their impact on TBC.

Further research is necessary on the impact of organizational conditions on TBC and diabetic patient outcomes, particularly in low income settings.

## Data Availability Statement

All relevant data is contained within the article. All datasets analyzed for this study are included in the article/supplementary material.

## Author Contributions

The overall concept of the project was devised by ML-P in collaboration with DD, RS, MG, and JN. The data collection and the analysis were performed by MG and ML-P. ML-P and MG collaborated on the development of the original final draft which was critically revised by DD, RS, and JN for important intellectual content. Comments were incorporated by ML-P. The authors certify that this or similar material has not been submitted or published in any other publication. The authors take full responsibility for the work.

## Conflict of Interest

ML-P was employed by the company Impactivo, LLC. MG was employed by the company Impactivo, LLC. The remaining authors declare that the research was conducted in the absence of any commercial or financial relationships that could be construed as a potential conflict of interest.
